# Primary headache syndromes in the elderly: epidemiology, diagnosis and treatment

**Published:** 2016-04-15

**Authors:** Willebrordus P. J. van Oosterhout, Carlo Cheung, Joost Haan

**Affiliations:** 1 *Department of Neurology, Leiden University Medical Center, Leiden, the Netherlands*; 2 *Department of Neurology, Medical Center Haaglanden, the Hague, the Netherlands*; 3 *Department of Neurology, Alrijne Hospital, Leiderdorp, the Netherlands*

**Keywords:** cluster headache, elderly, migraine, primary headaches, tension-type headache

## Abstract

Although secondary headaches due to e.g. temporal arteritis or a brain tumor are common among the elderly, primary headache disorders also occur in this age group, albeit less frequently than in younger individuals. A different presentation in the older age groups often makes a diagnosis difficult. Some headache syndromes, such as hypnic headache, are typical for the elderly. Furthermore, age-related physiologic changes, co-morbidities and contra indications require appropriate and targeted treatment in the elderly. Although treatments for the most common primary headaches are available, many limitations hamper their use in this group. For many headaches syndromes randomized controlled treatment trials in elderly are not available. In this article we review the clinical aspects of common primary headaches and medication overuse headache in the elderly and their treatments, with emphasis on age-specific problems.

**Relevance for patients:** Primary headache syndromes in older patients ask for specific treatment considerations due to comorbidity, polypharmacy and an increased risk of side effects. Clinically, the headaches can be different and atypical. Results from clinical trials cannot be generalized to the elderly because these groups usually are not included in studies. In migraine, non-pharmacologic treatment should be considered, with depression and cerebrovascular disease as major comorbidities. Tension type headache, being the most common headache presentation in elderly, also includes a relatively large proportion of patients with a secondary headache warranting further testing. Trigeminal autonomous cephalalgias are rare, and can present with pseudo dementia. Medication overuse and medication-induced headaches are relatively common, for which patient education, ceasing drugs and withdrawal from caffeine containing substances are pivotal. Furthermore, hypnic headache, exploding head syndrome and benign thunderclap headache are specific for this patient group and require specific treatment.

**This article is adapted and translated with permission from Tijdschr Neurol Neurochir 2015;116(4):174-179**.

## 1. Introduction

Primary headache disorders regularly occur in the elderly, albeit less frequently than in younger age groups, with reported prevalence ranging from 52%-81% [1,2]. It is difficult to give a proper definition of the ‘elderly patient’. A higher calendar age does not always reflect a higher biological age, and the studies addressed in this review have used different age cut-offs for defining elderly patients (with ages above 50, 55, 60 or 65 years). We therefore have chosen to use the age categories as described in the original studies.

The elderly population is increasing in size in Western society, which results in a higher absolute number of elderly patients with headaches. Specific knowledge on the treatment of primary headache syndromes is important; comorbidity, polypharmacy and an increased risk of side effects have to be taken into account when treating this group. In this review article, we will discuss the clinical characteristics of primary headache syndromes in the elderly, as well as treatment options, pitfalls and caveats. Although headaches induced by analgesics use suggest that these headaches are secondary, we have also included the medication overuse headaches, since it is unclear why analgesics do lead to headaches in headache patients, but not in patients with other chronic pain conditions.

## 2. Migraine

### 2.1. Clinical characteristics

Migraine attacks decrease in frequency or cease completely after the age of 50-60 years in the majority of both male and female patients [1,2]. In a substantial minority of patients over 60, however, migraine attacks persist, resulting in a high absolute number of elderly patients with migraine [1,3]. The incidence of new-onset primary migraine over the age of 60 is largely unknown. Only one study found that 0.5% of all new-onset headaches in patients over the age of 65 were migraine headaches (compared to 53% of new-onset headaches in the age group < 65 years) [4]. The lifetime prevalence of migraine in individuals over 55 years is 20-34% [3]. Clinically, migraine characteristics can change with increasing age. Attack severity decreases, throbbing or pulsating headaches become less frequent, and accompanying symptoms occur less often [5], which hampers distinguishing these migraines from tension-type headaches [6]. Remarkably, migraine headaches cease whereas aura symptoms persist in patients with migraine with aura [2,6,7]. In clinical practice, distinguishing between aura without (migraine) headache and a transient ischemic attack or minor stroke can be difficult. This is, however, of utmost importance since these conditions have different treatments [8]. Chronification of migraine can occur in the elderly too, both with and without medication overuse [9]. Furthermore, migraine attacks tend to start more often (up to 60%) in the night or early morning [10].

Comorbidities including arthritis, hypertension, (cardio) vascular conditions, diabetes, and malignancies are common in this group [2]. When treating migraine, cerebrovascular diseases and depression are particularly relevant [6]. Migraine seems to be associated with increased risks of stroke (both ischemic and haemorrhagic), heart disease, retinal vasculopathies and higher overall mortality [2,11]. In women aged 60-74 years active migraine was found more often in case of comorbid depression [12].

### 2.2. Specific treatment in the elderly

Ageing induces physiological changes in gastrointestinal motility and function, liver and kidney function, and vascular system and bodily composition, resulting in different pharmacodynamics and pharmacokinetics [2] and a higher incidence of side effects [13]. These need to be considered when treating the migraine, as well as interactions between anti-migraine and other drugs. Specific guidelines for the treatment of migraine in the elderly are not available. Treatment should therefore also be chosen pragmatically and on an individual patient level [6].

Pharmacological treatment can be used for both acute and prophylactic interventions. Simple analgesics are first choice when treating migraine attacks. It has been suggested to check transaminases and liver function in elderly patients who frequently use acetaminophen (over 3 grams/day) [14,15]. Acetylsalicylic acid can induce peptic ulcers and gastric haemorrhages. Antiemetics give a higher risk of extra-pyramidal symptoms as side effects. The use of NSAIDs is not advised in the elderly, as it increases the risk of dose-dependent gastrointestinal haemorrhages – specifically when combined with anti-coagulative therapies [16] – and mild renal failure, that can result in acute renal insufficiency, nephritis, proteinuria and edema. When in specific cases NSAIDs are prescribed, an additional proton pump inhibitor is recommended. One should be very precautious with opioids in the elderly because of sedative and cognitive effects, as well as constipation [2]. Preparations such as tramadol frequently induce nausea, confusional states or delirium [2,13]. There is no evidence nor consensus about the use of triptans as a migraine specific treatment in the elderly. In case of cardiac contraindications such as myocardial ischemia, triptans should not be used [17]. Based on expert opinions, a history of myocardial ischemia, however, is not an absolute contraindication for current use of triptans. There is no evidence that triptan use is associated with an increased risk stroke, myocardial infarction or cardiovascular disorder, not even in the group of elderly with cardiovascular risk factors [18]. In case of no absolute cardiac contraindication, the use of triptans can be considered reasonably safe. Consultation with a cardiologist is advised when in doubt. It can however be difficult to make an evidence-based choice.

When prophylactic treatment is considered, we advise to ‘start low and increase slow’. The different prophylactic drugs that are available tend to lead to more side effects in the elderly group than among younger patients [2,17,19]. Tricyclic anti-depressives (TCAs) are notoriously known for their anticholinergic effects such as sedation, a dry mouth, constipation and urinary retention [19]. Amitriptyline can induce orthostatic hypotension, including more chance of falling [2,17], but can also trigger epileptic seizures, cardiac conduction abnormalities (prolonged QT-time) and urinary retention (in case of benign prostate hypertrophy). Nortriptyline is usually better tolerated [17]. TCAs should not be used in patients with cardiac conduction abnormalities, closed-angle glaucoma or pre-existing urinary retention [2]. β-blockers have an increased risk of sedative effects, conduction abnormalities, asthma, glaucoma, depressive symptoms and diabetes. They are however probably safe to use in case of comorbid heart failure [17]. Valproic acid might induce liver enzyme disorders, bone marrow suppression, delirium, tremor, ataxia and in rare cases a pyramidal syndrome with dementia [2,17]. Topiramate also has many side effects, most often nephrolithiasis, weight loss, sedative effects or agitation. ACE-inhibitors and angiotensine-II-type-1-receptoragonists are a safer option of first choice that requires follow-up of kidney function [17]. Although intramuscular injections with botulinum toxin A (Botox®) have proven clinical efficacy in chronic migraine, the clinical benefit is very limited. Furthermore, patients over age 65 were not included in the clinical trials, thereby hampering extrapolation to elderly patients [20,21].

## 3. Tension type headache

### 3.1. Clinical characteristics

Tension type headache (TTH) is a very heterogeneous condition and the most common of all primary headache disorders. Three clinical subtypes can be distinguished: an episodic form (headaches on ≤ 1 day per month); a high frequency episodic form (headaches on 1-14 days per month) and a chronic form (headaches on ≥ 15 days per month) [22]. TTH usually is a pressing headache and feels like a band is strapped over the skull. Clinical characteristics in the elderly population are similar to other age groups. Since TTH is a featureless headache syndrome, other primary and secondary headache syndromes should always be considered or investigated in the elderly. In patients > 65 years, 20-30% of new headache symptoms are caused by underlying diseases. In comparison, in patients < 65 years, this proportion is only 1.3%. Therefore, history taking, physical and neurological examination should be aimed at detecting possible ‘red flags’ suggestive of the underlying pathology. However, in the majority of these patients > 65 years (50%-80%), a final diagnosis of TTH can be made [3].

TTH prevalence is approximately 25% among individuals 60-65 years of age [1,23]. Among those aged 65-96, overall TTH prevalence is comparable, although it is higher in the highest age groups [24]. 1.5% of men and 2.7% of women > 60 year suffer from chronic TTH [23], thereby making this a very common condition. TTH is a persisting condition: after 13 years of follow-up 27% of patients still had headache symptoms. Notably, these were all women, and without an additional medication overuse headache at baseline [25].

### 3.2. Specific treatment in the elderly

When treating TTH, one can choose pharmacological or non-pharmacological options, although the clinical efficacy of both is very limited [26]. Acetaminophen, acetyl salicylic acid, ibuprofen, naproxen, ketoprofen and diclofenac are considered ‘effective’ and caffeine is considered ‘probably effective’ according to European guidelines [27]. Acute attack treatment should be limited to 2 days per week (similar to younger patients) to avoid secondary medication overuse headaches. NSAIDs are most effective, with no evidence for superiority for a specific NSAID [28]. Side effects of NSAIDs are very relevant for the elderly (see also section on migraine). Preparations combining analgesics and caffeine are frequently used and might give additional pain relief [27]. Prophylactic treatment can be considered with >2-3 days of headache per week. Studies show that amitriptyline (up to 75mg/day in younger age groups) has the highest efficacy, especially in chronic TTH [29]. In elderly patients, titration needs to start low and increase slowly [30]. A combination of amitriptyline and the muscle relaxant tizanadine results in a higher clinical effect [31]. Nortriptyline, protriptyline, doxepin and mirtazapine have been studied less. It is suggested these have similar efficacy with less side effects [32]. In general, the use of antidepressants is limited by the side effects. TCAs should be started on a low daily dosage and increased slowly with careful monitoring. If patients simultaneously use selective serotonin reuptake inhibitors (SSRIs) for other conditions, even lower daily dosages are needed as SSRIs decrease TCA metabolism. Botulinum toxin A (Botox®) injections are not considered effective.

Especially in patients with high frequency episodic TTH there is a risk of chronification. Risk factors for chronification are analgesic overuse, a history of migraine, depression, other pain syndromes, obstructive apnea syndrome, and excessive use of caffeine [17]. It is advised to treat these modifiable risk factors, especially in the elderly patient since pharmacological options are limited due to drug-drug interactions and side effects.

## 4. Trigeminal autonomous cephalalgias

### 4.1. Clinical characteristics

Trigeminal autonomous cephalalgias (TACs) are relatively rare conditions in the elderly population. The most common is cluster headache (CH), followed by paroxysmal hemicranias, SUNCT, SUNA and hemicrania continua. Only few cases of the more rare TACs have been described, so little is known about the disease progression over time [6]. CH incidence is assumed to decline with increasing age, although precise data are lacking. It is suggested the chronic form of CH is more common in the elderly population with higher prevalence among women [6]. Symptoms of CH are similar to those in younger patients. Strong agitation, sometimes even presenting as a pseudo-dementia, can be the only accompanying symptom [33].

### 4.2. Specific treatment in the elderly

There is no evidence for the efficacy of non-pharmacological treatment strategies In general, however, avoiding alcohol during the bouts is advised. Acute treatment of CH attacks comprises oxygen inhalation and subcutaneous injection of sumatriptan. Oxygen inhalation is a safe option in the elderly patient. Prolonged inhalation should be avoided in patients with comorbid chronic obstructive pulmonary disease (COPD), as carbon dioxide levels can increase easily [17]. As previously mentioned, there is no contra-indication for the use triptans if cardiovascular risk factors are taken into account. Prophylactic options include verapamil and anti-epileptics (topiramate). These can be used safely, but again have a higher risk of side effects in this population. Verapamil interacts with antihypertensive drugs, simvastatine, atorvastatine and carbamazepine. Frequent side effects are constipation, dizziness, bradycardia, hypotension, peripheral edema and erectile dysfunction. Lithium has more severe side effects, especially in this group, including tremors, nausea, thirst and visual and speech disturbances. Hypothyroidism and diabetes insipidus have been described as well. Lithium should not be used in patients with heart or renal failure. Corticosteroids can be used as short-lasting prophylactic or bridging strategy. Prolonged use should be avoided since it leads to osteoporosis, diabetes mellitus, gastro-intestinal symptoms and can induce or exaggerate current or pre-existent psychiatric conditions. If methysergide is used, a one month drug holiday after every 6 months is advised to reduce the risk of retroperitoneal, pleural and pericardial fibrosis [17,34].

## 5. Thunderclap headache and other primary headaches

### 5.1. Clinical characteristics

Benign thunderclap headache (BTH) is a rare primary headache syndrome with an estimated life-time prevalence of 0.3% in the population 55-94 years of age [1]. Clinically it is characterised by a sudden and severe headache that reaches maximum intensity within 1 minute. Secondary aetiologies including subarachnoid haemorrhage (the most frequent cause), reversible cerebral vasoconstriction syndrome, arterial dissection, cerebral venous sinus thrombosis, pituitary apoplexy, intracranial haemorrhage and other causes should be excluded. Clinically it is difficult to differentiate between BTH and a secondary headache [35]. Other rare primary headache syndromes include primary cough, stabbing or exertional headache, and hemicrania continua. The estimated life-time prevalence of < 1.0% of these headaches is suggested to be higher in this age group compared to the younger population, although clear data are not available [1,3,34,36].

### 5.2. Specific treatment in the elderly

Benign thunderclap headache can be treated with acetaminophen, NSAIDs, or transiently opioids, taking into account the aforementioned limitations of opioid use in the elderly. For the other primary headache syndromes, prophylactic treatment with indomethacin, propranolol, topiramate, etoricoxib or lithium can be considered. The complete response to indomethacin is pathognomonic for hemicrania continua.

## 6. Medication-induced and medication overuse headache

### 6.1. Clinical characteristics

Medication overuse headache (MOH) and medication-induced headache are common conditions: approximately 15% of all headaches in patients over 65 years is induced or maintained by drugs [36]. This comprises both headaches as a side effect from non-headache medication, as well as headaches induced by analgesic use. Headaches are common side effects of prostaglandin synthetase inhibitors (indomethacin), dihydropiridines (niphedipin), H2-recepter antagonists (cimetidine; ranitidine), β-blockers (atenolol, metoprolol), sulphonamides (trimethoprim-sulfamethoxazole), nitrates (isosorbide dinitrate), ACE inhibitors (captopril), dipyridamole and α2-stimulants (methyldopa). On the other hand, chronic use of analgesics, including NSAIDs, aspirin, ergotamine, acetaminophen, caffeine, opioids, or codeine induces MOH.

### 6.2. Specific treatment in the elderly

Treatment is similar to younger age groups and is based on patient education, ceasing headache inducing or maintaining drugs and withdrawal from caffeine-containing food and beverages for three months. After this combined withdrawal, a possible underlying primary headache syndrome can be diagnosed and treated [13].

## 7. Hypnic headache

### 7.1. Clinical characteristics

Hypnic headache (HH), also known as ‘alarm clock headache’ is only seen in patients over 50 years of age, and comprises 0.07-0.35% of all headaches in this group. The pathophysiology has not yet been elucidated [37]. Characteristic for HH are attacks of headache, lasting 15-40 minutes, without accompanying symptoms. Attacks occur on > 10 days per month, during at least 3 consecutive months [21]. The headache initiates during sleep and wakes the patient. Many patients report comorbid migraine or TTH. Obstructive sleep apnea syndrome and nocturnal hypertension are often found as well [37].

### 7.2. Specific treatment in the elderly

It is not always necessary to treat HH, since the attacks are shortlasting and of mild to moderate severity. If patients experience longer episodes with nocturnal attacks, caffeine or caffeine-containing analgesics and triptans are the treatments of choice [37]. When treating prophylactically, caffeine, lithium or indomethacin ante noctem can be considered. As mentioned before, the use of lithium should be limited in the elderly patient because of side effects and contra-indications. Indomethacin increases the risk of gastro-intestinal haemorrhages and should be avoided in patients with renal failure. The efficacy of other prophylactic drugs such as topiramate, TCAs, melatonin, oxetorone, gabapentin, lamotrigine, pregabalin and acetazolamide has not been studied. These drugs should therefore not be used in the elderly patient.

## 8. Exploding head syndrome

### 8.1. Clinical Characteristics

Exploding head syndrome (EHS) is a benign condition occurring in patients over 50 years of age. Characteristically, patients get woken up by the perception of a sound or explosion in the head during sleep (30%) or during the sleep-wake transition phase (65%). Usually, there is no accompanying headache. EHS is considered a parasomnia and very little is known about the condition [38]. The sounds that patients describe range from ‘fireworks being set off’ to a ‘gunshot’, start abruptly and are shortlasting. When waking up, patients frequently feel anxious with tachycardia and excessive sweating. Visual light flashes and myoclonus are reported as well. EHS can occur monophasic, but multiple periods with multiple attacks per night are also reported [38]. Almost always attacks cease spontaneously, although sporadic recurrences after years have been described. There are no epidemiologic data on EHS; only 112 cases have been reported in literature so far with a male-to-female ratio of 1:1.55 [38]. EHS etiology is unclear. Sleep deprivation and stress could be triggering factors.

**Table 1.** Prevalence and treatment of primary headache syndromes in the elderly (> 55 years old).

Headache syndrome	Prevalence	Change in clinical presentation	Treatment		Caveat Always: more drug-drug interactions and side effects
			Attack	Prophylaxis	
Migraine	1-year: 6%-20% Life-time: 20%-34%	Headache severity decreases. Aura persists. Attacks more often in nights and mornings	Tailor-made. Consider non-pharmacologic treatment		Comorbid depression and cerebrovascular conditions
			Simple analgesics Triptans	Start low, increase slowly
Tension type headache	Episodic: 25% Chronic: 1%-3%	Similar to younger patients. Pressing, band feeling, no clear accompanying symptoms	Maximum 2 days/ week Simple analgesics +/- caffeine	Amitriptyline	20%-50% of TTH phenotype is secondary
Trigeminal autonomous cephalalgias	Rare	Relatively more often women. Little/no accompanying symptom	Oxygen Triptans	Avoiding alcohol in bout Verapamil; lithium; anti-epileptics	Pseudo dementia as presentation of agitation/urge to move
Medication-induced/overuse headache	15%	Related to drugs: as side effect / analgesics overuse	Education; withdraw all analgesics and caffeine/thein-containing drugs/ foods/ beverages. Afterwards: treat underlying primary headache syndrome		Frequent use of drugs with headache as side effects, or analgesics overuse
Hypnic headache	0.07%-0.35% of headache Almost all patients >50 years	Nocturnal attacks, 15-40 minutes, no accompanying symptoms ‘Alarm clock headache’	Not always necessary: shortlasting and mostly self-limiting. Option: caffeine +/– simple analgesics; triptans	Caffeine Lithium Indomethacin	Sleep apnea Nocturnal hypertension Comorbid migraine and TTH
Exploding head syndrome	N = 112 cases; all > 50 years	Male / female= 1 : 1.55 Attacks with perception of sound, fear and sometimes headache. During sleep (30%), or sleep/wake transition (65%).	Information; reassurance	TCAs; anti-epileptic drugs; calcium channel blockers (all without evidence) Treatment of comorbid sleep apnea	Considered a parasomnia Obstructive sleep apnea syndrome
Benign thundercla headache	0.3% (primary thunderclap)	Suggested to be more prevalent in elderly Acute onset, severe headache, sometimes accompanying symptoms	Simple analgesics	Not necessary	Exclude underlying intracranial pathology

### 8.2. Specific treatment in the elderly

Treatment consists of patient education and reassurance, as most patients fear a stroke [37]. Treating the common comorbid obstructive sleep apnea syndrome can reduce EHS attack frequency. Open and controlled clinical trials investigating drug treatment are lacking. In case reports TCAs (clomipramine 50 mg/day), anti-epileptic drugs (topiramate 200 mg/day; carbamazepine 200-400 mg/day), and calcium channel blockers (flunarizine 10 mg/day; nifedipine 90 mg/day) might reduce attack frequency and severity [38,39]. These drugs should nevertheless be used with precaution.

## 9. Conclusion

In this review we have discussed the clinical characteristics and treatment options for common and specific primary headache syndromes in the elderly patient. Clinical information is not widely available for some headache syndromes and treatment is mostly based on expert opinion or case reports rather than on evidence from clinical trials. Nevertheless, there are acute and prophylactic therapeutic options for these patients. Comorbidity, polypharmacy and increased risk of side effect need to be taken into account in every tailor-made treatment choice. The elderly population is increasing in size, and headache is a common condition. Therefore, it important that the treatment options are known and considered, since the scientific evidence and therefore clear guidelines are not available yet.

## 10. Directions for clinical practice

Migraine and trigeminal autonomous cephalalgias are common among the elderly although less common than in younger age groups, whilst hypnic headache has a higher prevalence;

Clinical presentation of migraine and trigeminal autonomous cephalalgias can be different and atypical in the elderly;

Tension Type Headache is the most common primary headache syndrome in the elderly population, but a secondary headache should always considered and excluded in this patient group;

The treatment of primary headaches in the elderly is tailor-made, and it is pivotal that the physiology of normal ageing, comorbidity, polypharmacy and increased risk of side effects are taken into account in acute and prophylactic therapeutic strategies.
